# Advances in Biodegradable Nano-Sized Polymer-Based Ocular Drug Delivery

**DOI:** 10.3390/polym11081371

**Published:** 2019-08-20

**Authors:** Courtney Lynch, Pierre P. D. Kondiah, Yahya E. Choonara, Lisa C. du Toit, Naseer Ally, Viness Pillay

**Affiliations:** 1 Wits Advanced Drug Delivery Platform Research Unit, Department of Pharmacy and Pharmacology, School of Therapeutic Sciences, Faculty of Health Sciences, University of the Witwatersrand, Johannesburg, 7 York Road, Parktown 2193, South Africa; 2 Division of Ophthalmology, Department of Neurosciences, School of Clinical Medicine, Faculty of Health Sciences, University of the Witwatersrand, Johannesburg, 7 York Road, Parktown 2193, South Africa

**Keywords:** polymers, nanotechnology, ocular drug delivery, biodegradable

## Abstract

The effective delivery of drugs to the eye remains a challenge. The eye has a myriad of defense systems and physiological barriers that leaves ocular drug delivery systems with low bioavailability profiles. This is mainly due to poor permeability through the epithelia and rapid clearance from the eye following administration. However, recent advances in both polymeric drug delivery and biomedical nanotechnology have allowed for improvements to be made in the treatment of ocular conditions. The employment of biodegradable polymers in ocular formulations has led to improved retention time, greater bioavailability and controlled release through mucoadhesion to the epithelia in the eye, amongst other beneficial properties. Nanotechnology has been largely investigated for uses in the medical field, ranging from diagnosis of disease to treatment. The nanoscale of these developing drug delivery systems has helped to improve the penetration of drugs through the various ocular barriers, thus improving bioavailability. This review will highlight the physiological barriers encountered in the eye, current conventional treatment methods as well as how polymeric drug delivery and nanotechnology can be employed to optimize drug penetration to both the anterior and posterior segment of the eye.

## 1. Introduction

The eye is one of the most well protected organs in the body. It is made up of many complex layers and structures, with a variety of defence mechanisms. These barriers, designed to protect the eye against foreign particles, molecules and infectious organisms, also greatly inhibit the movement of active ingredients into the eye. This presents a challenge when it comes to delivering drugs effectively to treat ocular conditions. Many of the formulations which are currently available on today’s market experience low bioavailability and rapid clearance from the administration site, resulting in a frequent dosing schedule [1]. The dosage frequency depends on the route of delivery; for example, liquid eye drop formulations are generally administered on a daily, if not 2–3 times a day, whereas intravitreal injections can be administered every 4 to 6 weeks [2,3].

The effective treatment of ocular conditions is of paramount importance. Many of these conditions, such as glaucoma (in which most therapies targets the anterior segment) and age-related macular degeneration (a posterior segment condition), negatively impact the patient’s vision. If these conditions are not effectively treated and the vision impairment prevented, the damage or vision loss is irreversible [2,3]. An impairment of vision can have devastating effects on a patient’s quality of life. They are restricted or unable to perform everyday tasks, thereby resulting in limited physical activities [4].

When considering the challenges that are faced in delivering drugs to the eye, many innovations are being made, employing both polymers and nanosystems in order to optimize this route of drug delivery. Polymers, specifically biodegradable polymers, offer a number of benefits when it comes to enhancing therapeutic ophthalmic formulations. The primary benefit is their mucoadhesive property, especially in the region of the cornea and conjunctiva. This allows for a formulation to have an increased residence time on the corneal epithelial surface which allows for improved drug penetration [5].

Nanoscaled drug delivery systems have been widely investigated in order to optimize ophthalmic therapeutic preparations. They provide a number of benefits, ranging from sustained drug release profiles to improved permeation through ocular barriers. Not only are they able to improve formulations used to treat the anterior segment of the eye, they are also able to improve drug delivery to the posterior segment of the eye, a region that is notoriously difficult to treat [6].

This review aims to provide an overview on recent advances in ocular drug delivery and limitations of the conventional delivery systems on the market. In doing so, the importance of biodegradable polymers and nanotechnology in this field of pharmaceutical development will be highlighted by illustrating current challenges faced in ocular drug delivery and how they can be exponentially modified for greater ocular bioavailability.

## 2. Ocular Physiological Defense Mechanisms for Drug Delivery

The first defence mechanism involves the structures anterior to the eye, such as the eyelid and lashes. These structures, along with the body’s blinking reflex, are able to defend the eye against intrusion of particles which could cause mechanical damage to the eye if they were to come into contact with it. This blinking reflex plays a role in the challenges faced by current ocular drug delivery systems by rapidly removing the formulation from the surface of the eye and thereby not allowing the drug adequate time to penetrate through the barriers, which will be described in detail below [7].

The eye is divided into two segments; the anterior segment (which encompasses the tear film, cornea, conjunctiva, iris, ciliary body, aqueous body, lens and anterior sclera) and the posterior segment (which encompasses the posterior sclera, choroid, Bruch’s membrane, retina and vitreous humor). There are a myriad of conditions which impact the anterior or posterior segments respectively and each of these requires treatment in one way or another. Although drug delivery to the anterior segment of the eye is not without its challenges, conditions which affect this segment of the eye are easier to treat because the active ingredients are able to be applied topically, which is easier and less invasive for the patient [8].

Drugs that are administered to the surface of the eye are removed via the nasolacrimal drainage system and are absorbed by the nasal mucosa. This not only prevents the active ingredient from entering the aqueous humor, but can also result in unwanted side effects as it enters systemic circulation [9].

Ocular active ingredients are also removed from the eye via efflux transporters. These transporters are ATP-binding proteins that are responsible for protecting cells by removing various substances such as drugs, metabolic compounds and lipids. The efflux transporters which are most prominently expressed in ocular epithelium are P-glycoprotein and multidrug resistant protein. Multiple studies have assessed that administering active ingredients that inhibit these transporters along with ocular active ingredients may help to improve the effectiveness of ocular drug delivery systems [10].

Ocular immune privilege was first described by Sir Peter Medawar when he observed that grafts, composed of tissues foreign to the eye, were not rejected when they were placed into the anterior chamber. It was initially thought that the immune privilege in the eye is due to the fact that there is no direct lymphatic drainage from the eye as well as the presence of the blood-ocular barrier, which prevents the entry of substance into the aqueous humor from the systemic circulation [11]. However, it has been reported that this process is much more complex, as other factors such as the presence of immunodulatory factors (namely AqH) and antigen-presenting cells (APCs) play a significant role. The quality of immune privilege allows for the eye to have an immune response to a foreign pathogen, without causing damage to local tissues which is usually an effect of an immune response and the inflammatory process that follows. This allows for foreign substances such as ophthalmic formulations to be administered to the eye and enter both the anterior segment and the posterior segment without loss of the specialized tissue cells responsible for the sense of sight [12].

**Figure 1:**
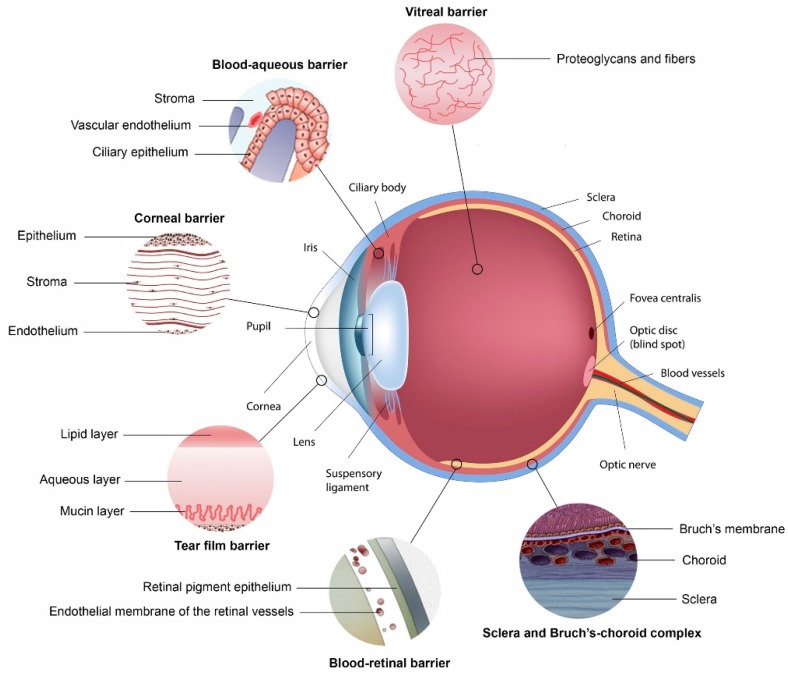
Ocular physiological defenses that provide barriers for the delivery of drugs to segments of the eye, with each barrier highlighted, preventing drugs from penetrating across each membrane (Reprinted with permission from [13]).

This review will highlight how each of the main ophthalmic barriers to ocular drug delivery can be overcome with the use of polymeric nanosystems. Figure 1 illustrates structures within the eye that hinder the absorption of administered drug. Many of these act as a barrier to the delivery of drug to the eye, as discussed below. The two blood-ocular barriers are also illustrated [13].

### 2.1. Cornea

The cornea forms one of the major barriers for the transport of substances into the eye. This barrier, which is made up of multiple layers of tissue (the corneal epithelium, Bowman’s layer, the stroma, Dua’s layer, Descemet’s membrane and the endothelium), is kept moist by tears. Lacrimation and tear turnover are also responsible for flushing away any matter which is foreign to the eye, including a large quantity of an administered formulation [14]. The tight cell junctions within the cornea prevent molecules from moving into the eye [15]. Due to the lipophilic properties of the corneal epithelium, hydrophilic molecules are largely unable to permeate through it [13]. The washing away of formulations which are applied to the eye is largely due to the rapid turnover rate of lacrimal fluid. This results in the active ingredient being removed from the surface of the eye, where it would permeate through the above described layers, to the nasolacrimal duct in only a matter of minutes [13].

Polymeric nanosystems are able to adhere to the mucosa of the eye, leading to an increased residence time at the cornea. This, along with the small particle size of nanosystems, allows for increased drug permeation across the cornea [16].

### 2.2. Conjunctiva

The conjunctiva is comprised of a thin membrane which is responsible for the production of tears as well as the maintenance of the tear film. There is also a rich supply of vasculature, such as capillaries and lymphatic drainage within the conjunctiva, resulting in the removal of active ingredient molecules [17]. These blood vessels do not contain tight cell junctions, so drugs that are administered locally are able to enter systemic circulation. This entry of active ingredients into the blood stream via the conjunctiva can lead to systemic side effects when the formulation is administered to the eye [1,13].

Polymeric nanosystems are able to provide mucoadhesive properties which prevents drugs from being removed by these mechanisms within the conjunctiva.

### 2.3. Sclera

The sclera, a collection of collagen fibres and proteoglycans, is both part of the anterior and the posterior segment of the eye. However, it is thicker towards the posterior segment, and responsible providing mechanical support thus for giving the eye its shape [1]. The permeation of drug molecules through the sclera is determined by the molecules’ hydrophobicity (the more lipophilic the molecule, the less it diffuses through) and charge (positively charged molecules bind to the proteoglycan matrix within the sclera and are unable to diffuse through) [17]. The sclera has been considered a viable option for the delivery of active ingredients to the posterior segment of the eye due to the fact that it has a large surface area, is accessible and is relatively permeable [18]. Recently, a study was conducted to develop and investigate polymeric particles that are able to be guided by magnetic forces across the sclera. This resultantly prevented the drug from being removed via bulk fluid flow from the posterior segment of the eye [19].

### 2.4. Blood-Ocular Barriers

There are two blood-ocular barriers which largely prevent the use of systemically administered active ingredients to treat ocular conditions, namely the blood-aqueous barrier and the blood-retinal barrier, as detailed in Figure 1. Although some active ingredients are able to pass through these barriers (such as those with high lipophilicity or with the aid of an active transport system), such a small volume of blood flows through the vasculature within the eye that a high dose would be needed, leading to systemic side effects [20,21].

### 2.5. Posterior Segment

Treatment to the posterior segment of the eye often includes invasive procedures such as surgery, implantation of a drug loaded device or injectable therapy (either intravitreally or subconjunctivally), due to the fact that active ingredients that are topically applied are not able to reach the vitreous humor in effective concentrations [18].

The retina forms part of the posterior segment of the eye. It is responsible for our ability to see. Diseases affecting the retina include age-related macular degeneration and diabetic retinopathy [18]. It forms part of the blood-retinal barrier. Delivery of drugs to the retina is challenging and could benefit greatly from the development of polymeric nano-drug delivery systems [22].

Each of these barriers plays a role in limiting the entry of active ingredients into the eye, leading to the low bioavailability of many ophthalmic formulations.

## 3. Current Limitations of Conventional Delivery Systems Employed for Ocular Therapeutics

Ophthalmic conditions and diseases are treated using a variety of applicable administered methods. Each of the methods currently on the market has its own limitations, advantages and disadvantages both in terms of the delivery of the drug, and also from a patient’s compliance perspective. A summary of these are provided below in Table 1.

### 3.1. Eye Drops (Solutions and Suspensions)

The current first line treatment for many ocular conditions, particularly those affecting the anterior segment of the eye, is eye drops. However, traditionally, these formulations have a low bioavailability and a rapid clearance from the administration site due to the defence mechanisms, as highlighted above. Researchers have tried to enhance the bioavailability of topical eye drop preparations in a number of ways, such as the addition of penetration enhancers and viscosity modifiers [21].

Due the direct administration of drops to the eye, it is important to consider the limit to dosage size that the cul-de-sac of the eye can withstand; usually between 7 and 10 µL. It has been shown that approximately 10% of an administered dose of eye drops (approximately 50 µL) will penetrate into the eye and reach the target site at an effective concentration. This often leads to a frequent dosing schedule [20,23]. The frequency at which some eye drops have to be administered have been shown to cause damage to the precorneal film of the eye. Benzalkonium Chloride (BAK), a quarternary ammonium, is the most commonly used preservative in eye drops. It acts as a detergent, and although highly effective against bacteria, it damages the lipid layer of the tear film. BAK has also been implicated in decreasing the number of goblet cells, leading to reduced mucin production and tear film instability. It also has a direct, dose dependent toxic effect on the corneal epithelium and has been shown to reduce the number of microvilli on the epithelial surface. All of these changes cause causing evaporative dry eye [24].

Although there are challenges when it comes to topical ocular drug delivery, which is a drawback to this method of treatment, it must be noted why it is one of the primarily used ocular treatment regimens. Topical ocular preparations are able to be self-administered and are non-invasive for the patient [21]. It is thus understandable why a patient would opt for topical treatment rather than another option which is more invasive.

However, patient compliance to eye drops is low. One must consider the inconvenience of daily eye drop administrations in patients with chronic ocular conditions, such as glaucoma. Research has shown that a large number of patients, who are supposed to be on treatment indefinitely, stop receiving their medication after a period of time and discontinue treatment [25,26]. A study has shown that the adherence rate of patients who are using eye drops for the treatment of glaucoma ranges between 30% and 80%. The reasons for the low level of adherence, according to the literature, range from a lack of understanding for the need to administer treatment regularly, to the cost of medication. Other reasons include patients simply forgetting to administer it [27].

Eye drops allow the patient to administer their medication themselves at home. However, there are limitations to this. As previously described, due to the low bioavailability and rapid clearance of these formulations, patients often have frequent dosage regimens. This further decrease patient adherence. Patients also need to be educated on the correct way to use and store their medications in order to prevent the spread of infection from either from one eye to the other, or from one patient to another.

The use of both polymers and nanosystem can be employed in optimizing eye drops to have an increased residency time, improved permeability into the target site and better bioavailability. This will lead to less frequent dosing schedules, for example, changing a dosing regimen from two or three times a day administration to daily or weekly administration, will in turn help to improve the rate of patient adherence.

### 3.2. Ointments

Ointments for the delivery of ocular active ingredients have been developed extensively to date. Due to the viscous nature of the formulation, they are not washed away from the eye as rapidly as liquid formulations, resulting in a higher bioavailability. However, this viscosity also leads to temporary blurred vision and inaccurate dosing [20]. For this reason, white petrolatum is often used as an ointment base. It has a suitable melting point which will result in a decreased viscosity once it has been administered to the eye [28].

Although ointments are advantageous in terms of bioavailability, they are not investigated as extensively as other ophthalmic formulations. This could be due to the fact that they have a number of formulation challenges such as poor content uniformity and reproducibility [28].

### 3.3. Intravitreal Injections

Injections directly into the vitreous humor are often used for the delivery of drugs to the posterior section of the eye. For example, anti-Vascular Endothelial Growth Factor (VEGF) drugs are primarily administered intravitreally for the treatment of age-related macular degeneration (AMD) and diabetic macular oedema. However, besides the fact that the procedure is invasive and unpleasant for the patient, these injections carry a number of risks. Major risks include possible endophthalmitis or retinal detachment, among others. These injections also have to be administered fairly frequently (for example, in the case of AMD treatment, every 4 to 6 weeks), resulting in poor patient compliance. Thus, the sustained drug release benefits that are found in nanostructures, further employing polymeric ocular drug delivery, could be utilized to extend the frequency at which these injections are to be administered [29,30].

### 3.4. Intraocular Implants

Another form of ocular drug delivery that has been developed are intraocular implants. Intraocular implants are surgically inserted into the eye where they release the drug over an extended period of time. Initially, these implants were not biodegradable and had to be surgically removed, which portrayed a number of risks associated with this application. However, the investigation of biodegradable polymers in the development of ocular implants transformed the application for surgical removal [29]. Implants are also able to be engineered as stimuli-responsive delivery systems. Currently, implants that are on the market, while being able to deliver drugs over a long period of time, are not able to change the rate at which they release drug. The investigations into stimuli-responsive implants is largely due to the developments which have been made employing stimuli-responsive polymers [31].

### 3.5. Contact Lenses

For more than a decade, contact lenses have been investigated for the use in ocular drug delivery. In contrast to eye drops, contact lenses are able to offer an increased residence time, allowing for improved drug delivery. However, it has been shown that contact lenses release the majority of the drug over the first few hours [32].

This drug delivery system works by diffusing the active ingredient out of the lens matrix, where it is able to come into contact with the surface of the eye, allowing for permeation [33,34].

However, the use of contact lenses as a drug delivery carrier system does pose risks. Patients must be informed on the correct, hygienic procedure when it comes to placing the lenses on the eye as well as taking them out in order to prevent the risk of infection. Sleeping with contact lenses in has been documented to lead to keratitis [33]. Thus, by developing nanoparticulate drug-loaded polymeric biodegradable lenses, the above limitations will be overcome, achieving site specific targeting over a controlled period of time.

### 3.6. Emulsions

There are a number of ocular emulsions currently on the market. The most common form of emulsion currently used for drug delivery is an oil in water emulsion, due to the fact that it is better tolerated by the eye than a water in oil emulsion. Emulsions have been shown to increase the bioavailability of an active ingredient by improving the residence time and permeation through the cornea. A study using azithromycin as an active ingredient also showed a sustained drug release profile, in comparison to an azithromycin suspension [35].

However, ocular emulsions are unstable and susceptible to flocculation. They are also destabilized by tear fluid [36].

By employing nanoemulsion techniques, this problem can be circumvented, enabling uniform dispersity within both phases of the emulsion formulation, delivering the drug in controlled pharmacokinetic profiles.

## 4. Biodegradable Polymers in Ocular Drug Delivery

Polymers, both synthetic and naturally occurring, have been used for a number of applications in the medical field, including; improved drug delivery, 3-D printing and tissue engineering, attributed to their biocompatible nature. Polymers are being considered more effective for pharmaceutical formulations, as they are able to improve the dosing of a particular active ingredient. They are also able to lower the side effects experienced by a patient by allowing implantation into the diseased tissue, thus providing increased drug loading at the site while reducing the systemic concentration [37].

In ocular drug delivery, polymers are widely accepted as being able to optimize a formulation through their mucoadhesive properties. This prolongs the time that the formulation is in contact with the cornea and conjunctival epithelium, helping to alleviate the challenge of rapid clearance from the eye which is often experienced by topical ocular formulations [38].

Certain polymers have been shown to have viscosity modifying properties, in addition to their mucoadhesive properties. When a polymer is being included in a formulation for its ability to increase viscosity, it is important to consider that if a solution becomes too viscous it will cause irritation to the eye. This prompts a defensive reaction in the eye, further increasing the production of tears and subsequent clearance of the formulation. It has been suggested that polymers should be added to a formulation in order to increase mucoadhesive without drastically altering the viscosity [38].

A further benefit to the use of certain polymers is their ability to biodegrade. This means that, once they have been administered, they are broken down by the body in non-toxic components. As the polymer is broken down the drug is released, resulting in sustained drug release profiles. The ability to biodegrade allows for a drug delivery system to be administered without the need for manual removal. This is of particular interest in the development of ocular implants, as it removes the need for surgical removal of the implant [39].

Certain polymers have been shown to be stimuli-responsive, allowing them to react or release an active ingredient upon a change in conditions, such as changes in temperature, pH or pressure. These are also known as “smart” polymers, and have been used in situ as gelling systems; formulations that undergo a change in viscosity as a response to a change in physiological conditions. This process allows the formulation to remain at the site of administration longer, thus allowing more time for the active ingredient to permeate through the cornea [40,41].

Bioresponsive polymers themselves are able to undergo a number of changes in response to a stimuli. These include changes in permeability, shape changes or phase separation, among others. This allows for the drug to be released from the formulation when it is needed [42]. The benefits of bioresponsive polymers can be seen in the developments that are being made in the field of ocular drug delivery. For example, implants developed to respond to inflammatory responses within the eye, have been investigated by many researchers to date [43].

**Figure 2:**
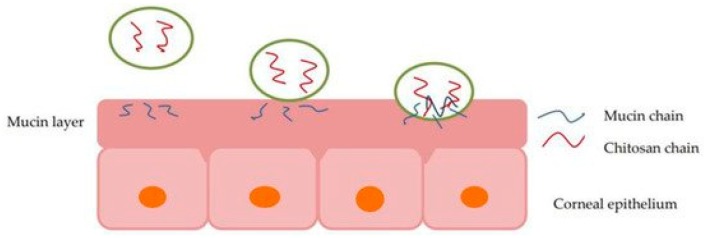
Illustration of chitosan interaction with the mucin layer of corneal epithelium, allowing particle permeation via mucoadhesion [45].

Although many new formulations are being developed using nanotechnology, polymers are also being used to enhance or optimize older formulations. It has been shown that by adding polymers with mucoadhesive or viscosity modifying properties to an eye drop formulation, it is retained at the site and thus increases the bioavailability of the drug [40,44]. Figure 2 depicts how a polymer, specifically chitosan, interacts with the mucin layers in the eye, reacting to mucoadhesive properties in situ [45].

A combination of polymers is often used in a single formulation. This is done in order to overcome one or more disadvantages that a certain polymer may have. For example, when used in a hydrogel, chitosan has been shown to have poor mechanical strength and low elasticity. However, when another polymer is added, such as poly(vinyl alcohol) (PVA), a more suitable hydrogel is formulated [44].

Many of the polymers discussed below are able to form polyelectrolyte complexes (PECs). These are formed when a positively charged polymer (such as chitosan) interacts with a negatively charged polymer (such as alginate) to form a cross-linked system. These complexes are then able to be utilized in the formation of nanoparticles and employed in drug delivery [45].

**Figure 3:**
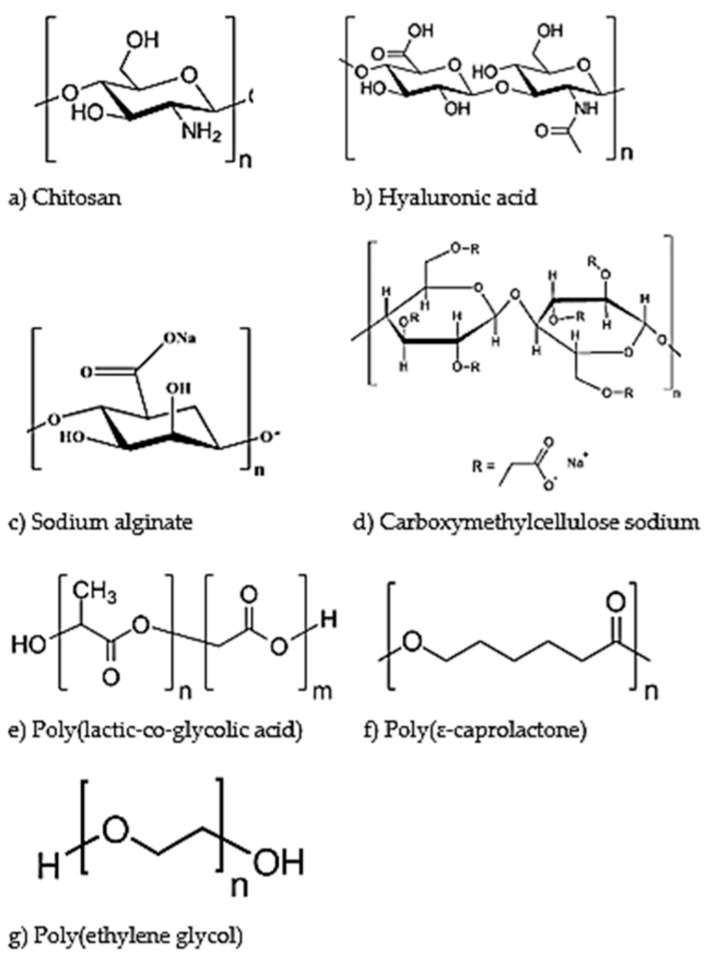
Chemical structures of biodegradable polymers employed for ocular drug delivery. The natural polymers discussed are chitosan (**a**), hyaluronic acid (**b**), sodium alginate (**c**) and carboxymethylcellulose sodium (**d**). The synthetic polymers discussed are poly(lactic-co-glycolic acid) (**e**), poly(ɛ-caprolactone) (**f**) and poly(ethylene glycol) (**g**).

The figure below (Figure 3) illustrates the chemical structure of each of the polymers (both natural and synthetic), as discussed in more detail below.

### 4.1. Natural Polymers

#### 4.1.1. Chitosan

Chitosan, which is derived from chitin, is often used as a polymer in ophthalmic preparations (chemical structure seen in Figure 3a). It is a cationic polysaccharide, allowing it to react with the negative charges found within the mucus and conjunctiva of the eye. Although chitosan has a number of beneficial properties, it is only soluble in acidic mediums, which would cause irritation if placed into the eye without being completely neutralized. This has led to the development a number of derivatives, such as galactosylated chitosan and thiolated chitosan, which have more favorable solubility profiles for ocular drug delivery [46,47].

Chitosan has a number of favorable characteristics; it is biocompatible, mucoadhesive, non-cytotoxic, as well as biodegradable [48,49]. It has been shown to increase the retention time of the formulation once it has been administered, as well as improve the penetration of the drug through the cornea by opening the tight cells junctions that are present within the epithelial tissue, as seen in Figure 2 [44]. The effect of chitosan on tight cell junctions and subsequent increase in permeability was shown using Caco-2 cells [50].

Chitosan has also been used in a number of developing ocular formulations because it has inherent antimicrobial and wound healing properties [51]. A modified derivative of chitosan, chitosan-N-acetylcysteine (C-NAC) has been shown to increase the rate of corneal healing in New Zealand White (NZW) rabbits when administered as eye drops twice daily [52]. The antimicrobial properties are derived from chitosan’s positive charge, allowing it to inhibit microbial growth through binding to the membrane wall of the microbe thus causing permeability changes [53].

This polymer, and its derivatives, is being developed into a number of nanotechnology formulations to treat a range of ocular conditions, such as nanoparticulate systems to treat glaucoma, conjunctivitis as well as multiple immune related ocular degenerative conditions [51,52,53].

#### 4.1.2. Hyaluronic Acid

Hyaluronic acid is a natural polymer found in numerous sites within the human body including the eye (chemical structure shown in Figure 3b) [54]. It has been investigated for use in ocular drug delivery systems because it is biocompatible, biodegradable and mucoadhesive. It is often used in combination with chitosan or other polymers [55]. A report by de la Fuente et al. highlights how hyaluronic acid can be used in conjunction with chitosan in a nanoparticulate drug delivery system for the administration of ocular gene therapy to both corneal and conjunctival cells [56].

Hyaluronic acid is a negatively charged polysaccharide. Like chitosan, hyaluronic acid is often modified and its derivatives used not only in drug delivery, but also other aspects of the medical field, such as tissue engineering [57]. It has been used to prolong the retention time of many ocular active ingredients such as timolol, pilocarpine and gentamycin through its mucoadhesive and viscosity modifying properties. Hyaluronic acid also acts as an active ingredient in certain formulations for the treatment of dry eye disease or artificial tears [57,58].

Hyaluronic acid is able to be functionalized in order to modify its properties in the formulation. This is possible with compounds such as tyramine [59], adipic dihydrazide (ADH) and methacrylic anhydride [60]. These researchers then formulated the functionalized hyaluronic acids into a respective hydrogel. These hydrogels could then be loaded either with a drug on its own, or with liposomes containing the drug (known as hyaluronic acid-based nanocomposite hydrogels). Through these drug delivery systems, researchers were able to obtain sustained drug release profiles. Thus, along with hyaluronic acid’s biocompatible and biodegradable properties, studies have demonstrated optimization of drug delivery to the eye achieving significantly improved pharmacokinetic properties [59,60].

Recently, hyaluronic acid has been used to formulate micelles, which were shown to be able increase the permeation of lipophilic ocular actives such as dexamethasone as well as reduce the rapid clearance rate from the eye [57].

#### 4.1.3. Sodium Alginate

Alginates are naturally occurring anionic polysaccharides that are largely used for their gelling abilities, allowing for viscosity modification when used in ocular formulations. They are derived from the cell walls of brown algae, as well as bacterial strains [61].

Sodium alginate is often used in ocular preparations due to the fact that is biocompatible, biodegradable, and allows for enhanced permeation (chemical structure shown in Figure 3c). However, it is very susceptible to enzymatic degradation. It is possible to modify sodium alginate using other polymers (such as poly(lactic-co-glycolic acid) (PLGA)) in order for it to function as desired [62].

Sodium alginate has been employed largely due to the fact that, in addition to its ability to alter the viscosity of a formulation, it is mucoadhesive and allows for sustained drug release. This helps to overcome some of the challenges experienced by topical ocular formulations [63,64].

Costa et al. reported the formulation of chitosan-coated alginate nanoparticles as a possible ocular delivery system for daptomycin. The researchers reported that the addition of sodium alginate to a nanoparticle formulation allows for a better sustained drug release profile than nanoparticles that only contain chitosan [65].

#### 4.1.4. Carboxymethylcellulose Sodium

Carboxymethylcellulose sodium (CMC) is a natural polymer that has been shown to possess thermoresponsive properties (chemical structure shown in Figure 3d). It is currently used in formulations to treat dry eyes [58,66]. Cellulose-derivative polymers are often used in ophthalmic preparations, due to their viscosity modifying abilities. Methylcellulose in particular has been shown to have ocular wound-healing properties, as well as the ability to act as a tear substitute [5,66].

Jain et al. reported the formulation of a polymeric membrane composed of sodium carboxymethylcellulose and polyvinyl alcohol (PVA), a water-soluble polymer that has been previously used in ophthalmic formulations. Sodium carboxymethylcellulose allowed for the formulation to have mucoadhesive and biodegradable properties, while the addition of PVA improved the rigidity of the membrane. These inserts could have applications in the sustained delivery of drugs to the eye [67].

### 4.2. Synthetic Polymers

#### 4.2.1. Poly(lactic-co-glycolic acid)

Poly(lactic-co-glycolic acid) (PLGA) is a synthetic, biodegradable polymer (chemical structure shown in Figure 3e). It has been largely investigated for drug delivery, tissue engineering and biodegradable sutures. This is mainly due to its biocompatibility and sustained release profiles as well as its ability to degrade in an aqueous medium. It has been formulated into nanostructures using a number of methods, including solvent evaporation and nanoprecipitation. PLGA can also be modified with the use of copolymers, such as polyethylene glycol (PEG), in order to enhance its characteristics. This has been shown in a study conducted by Vasconcelos et al., employing ocular drug delivery for sustained release kinetics of PLGA-PEG nanoparticles. These nanoparticles were conjugated with a peptide and provided a promising ocular drug delivery application, due to their low toxicity, sustained drug release and high entrapment efficiency profiles [68].

PLGA has been documented to also be used in the development of a biodegradable implant which is capable of delivering dexamethasone to the posterior segment of the eye. Studies have also investigated using PLGA nanoparticles, capable of delivering drug to the posterior segment of the eye after topical administration [69]. Thus, the use of PLGA, conjugated to other polymers, has to date significant applications in ocular drug delivery, ranging from simply improving drug permeation to increasing the residence time of nanoparticulate systems.

#### 4.2.2. Poly(ɛ-caprolactone)

Poly(ɛ-caprolactone) (PCL) is a synthetic polymer that has been commonly used in drug delivery due to its high biocompatibility and biodegradability (chemical structure shown in Figure 3f). It has been used in developments in ocular drug delivery, due to its ability to prolong drug release profiles [70]. PCL is able to be formulated into thin films as well as polymer solutions with low toxicity and slower degradation than that of PLGA [71].

It has been used in drug delivery systems such as implants that are able to release the active ingredient over a period of four months or more. PCL was formulated into a micro episcleral film, which was able to deliver triamcinolone acetonide over a four-month period, preventing the development of proliferative vitreoretinopathy after intraocular surgery or trauma [72].

Investigations into PCL use have shown that when it is used in conjunction with nanoscale drug delivery, such as nanoparticles, it has demonstrated greater concentration of both indomethacin and cartelol in the aqueous humor [73]. Da Silva et al. have also reported the development of dexamethasone acetate loaded poly(ɛ-caprolactone) nanofibers, which could be administered intravitreally post-surgery [74].

#### 4.2.3. Poly(ethylene glycol)

Poly(ethylene glycol) (PEG) is a non-ionic, synthetic polymer with hydrophilic properties (chemical structure shown in Figure 3g). It has been used in the development of ocular drug delivery formulations, both on its own and in conjunction with other polymers such as PLGA. The addition of PEG to a nanoparticle formulation has been shown to increase their mucoadhesive properties [68]. An example of PEG being used in combination with other polymers is a report by Shi et al., wherein a nanosuspension composed of methoxy poly(ethylene glycol)-poly(ɛ-caprolactone) and chitosan was formulated containing diclofenac. The study showed improved pre-corneal retention as well as penetration of the formulation across the epithelial membrane, resulting in a higher concentration of diclofenac entering the aqueous humor than that seen with commercial eye drops [75].

PEG has been used to enhance the performance of nanoparticles by increasing their permeation abilities. It also helps to prevent aggregation of nanoparticles, thereby improving their stability [76].

**Figure 4:**
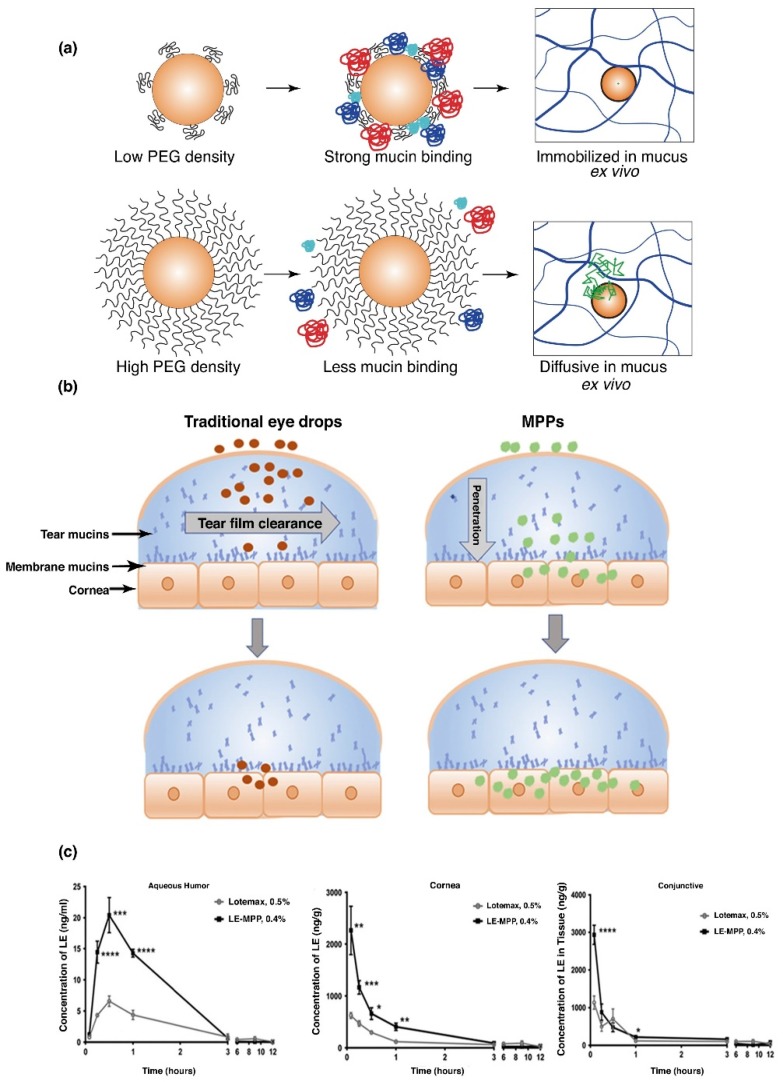
Poly(lactic-co-glycolic acid) polyethylene glycol (PLGA-PEG) nanoparticles, demonstrating greater mucin binding, employing lower density PEG coating compared to higher density, which decreases adhesion on the surface of mucin of the polymeric system (**a**). In vitro evaluation of PLGA-PEG nanoparticles, illustrating rapid diffusion in mucus ex vivo (**b**). Comparative analysis of commercial Loteprednol etabonate (LE) suspension eye drops to the polymeric delivery system in a New Zealand White (NZW) rabbit model, with almost 3-times greater Cmax profiles in rabbit aqueous humor, cornea and conjunctiva (**c**) (Reprinted with permission from [77]).

PEG was developed into a lipo-polymeric nanoparticulate system for the delivery of ketoconazole. This allowed for increased permeation of ketoconazole through the epithelia of the cornea resulting in an improved bioavailability [77]. Another study reported lower particle elimination through tear clearance, employing mucus penetrating particles of PEG-PLGA. A low PEG density was found to significantly bind to mucin, compared to the high density PEG, which demonstrated much lower binding affinity (Figure 4a). Drug KPI-121, formulation of 0.4% loteprednol etabonate (LE), coated with Pluronic (F127), significantly improved pharmacokinetic properties in a NZW rabbit model, in comparison to Lotemax1 0.5%, a commercially available suspension of LE (Figure 4b). It was further noted that KPI-121 (0.4%) of a single topical administration, increased Cmax of the drug by 3-fold, quantified in aqueous humor, conjunctiva and the cornea, when evaluated against the commercial product (Figure 4c). Bioavailability analysis further proved double potential of the polymeric delivery system, against the commercially available LE product, in respective ocular tissues of the cornea, conjunctiva and in aqueous humor [77]. Thus, the application of employing mucus penetrating polymeric platforms have great potential in increasing ocular bioavailability in the aqueous humor, cornea and the conjunctiva. Polymeric delivery systems, such as PEG, consequently will increase patient compliance by decreased dosing frequencies, portraying significant retention time in ocular tissue, especially the conjunctiva, which has more than 5-times greater surface area than the cornea, allowing mucin to retain greater concentration of the administered drug.

## 5. Nanotechnology Employed in Ocular Drug Delivery

Nanotechnology, or more specifically, nanosystems, have been used more extensively in the medical field in recent years. It has a number of applications, including improving ocular drug delivery. These nanosystems have been shown to increase the bioavailability of ocular active ingredients; the primary challenge faced by conventional ocular treatments. The intrinsic design and make up of nanosystems also allows for the protection of molecules, improved permeability through tissues and membranes and controlled drug release profiles [78].

Nanotechnology is defined as the development of structures and material which, in at least one dimension, fall within the nanometer scale [79].

One of the major benefits of employing nanosystems in ocular drug delivery are their ability to adhere to ocular tissue, mucosa and epithelium surrounding the eye; preventing formulations from being almost immediately washed away by the eye’s defence mechanisms. This capacity for mucoadhesion has been studied since 1985, and since then, many different types of nanotechnology, ranging from nanoparticles to nanowires, administered through numerous methods, from topical application to intravitreal injections, have been investigated and developed in order to optimize and improve the delivery of drugs to the eye [25]. The mucoadhesive property can be further advanced by the use of polymers in the formulation of the nanosystem. Increased mucoadhesive properties has been shown to increase the bioavailability of the active ingredient [80].

Until recently, nanosystems have largely been used to improve upon formulations which have already been developed and used on the commercial market. However, they are now been considered for formulations using active ingredients that are biologically active, but not able to be made into suitable formulations with conventional methods. Nanosystems can be used for targeted drug delivery as well as triggered release of active ingredients [81].

It has been highlighted by Weng et al. that, although nano-sized ocular drug delivery systems have the potential to overcome the problems that are faced by current commercial products, there is still research that needs to be done in order to fully optimize these systems. For example, many of the studies done on nano-sized drug delivery have included in vitro studies and not in vivo. Those studies that do include in vivo, employ a rabbit model, as their eye closely emulates those of humans. However, there are differences between the two, especially regarding surface sensitivity, mucus productions and tear production. Thus, these factors could lead to differential results when tested in humans [82].

When considering the safety of nanotechnology for ocular drug delivery, it is fundamental to consider the material that the nanoparticles are composed of, thus playing a crucial role in whether the system will cause irritation to the eye or not [82,83]. This also applies to the chemicals that are used during the formulation of the system. For example, in a study by Leonardi et al., it was shown that the surfactants largely employed as stabilizing agents in nanosystems cause irritation to the eye. This study showed that some surfactants caused more irritation to the eye than others and some only caused irritation when they were used above a certain concentration. Their results showed that Kolliphor^®^ P188 showed no irritation up to the highest concentration that was tested, Tween^®^ 80 did not cause irritation up to a concentration of 0.05% and sodium dodecyl sulphate caused severe inflammation. This study highlights the importance of both the removal of excess surfactant once the nanosystem has been formulated, as well as proper selection of a suitable surfactant [81].

There are many advancements being made in the use of nanotechnology in ocular drug delivery, which are aimed at reducing the challenges of low bioavailability. The following subsections will be aimed at providing applications of different nanosystems for improved delivery of drugs to the eye.

### 5.1. Nanogels

Nanogels are composed of polymers, either naturally occurring or synthetic, crosslinked to form hydrogel particles that are within the nanoscale. The selection of polymers employed, as well as the respective concentrations at which they are used, determines 14 of the characteristics of the resulting nanogel. These characteristics include, among others, the charge, hydrophilicity and softness [84,85].

Nanogels are able to be used for ocular drug delivery due to their high drug loading capacity. Due to the gelling nature and large surface area, they are able to adhere to the mucosa surrounding the eye, allowing for improved delivery of ocular active ingredients [86].

These drug delivery systems are able to be administered to the eye in the form of drops, making them a suitable patient-friendly treatment option [25]. This is due to the fact that nanogels are able to be engineered as in situ gels, whereby they can form a gel after application to the eye, as a response to a stimulus [87,88,89].

A study was performed by Liu et al., where an in situ nanogel system was developed for the delivery of curcumin, a compound which has low solubility and poor bioavailbility profiles when administered to the eye. The study showed that the nanosystem, compromised of a curcumin cationic nanostructured lipid carriers within a nanogel, had an increased mean residence time (MRT) within the aqueous humor and improved cornea permeation than that of a curumin solution [89].

When considering the use of nanotechnology in ocular drug delivery, the advancements that are being made aim to reduce, if not eradicate, the challenges faced by currently available ocular formulations.

### 5.2. Nanoparticles

Nanoparticles have a wide variety of uses, across a number of fields. In regards to the medical field, they have been extensively investigated and developed to aid in both diagnosis and treatment of diseases. This is largely due to the ability of nanoparticles to be engineered and subsequently functionalized in order to suit the needs of the system into which they will be placed [53,90]. There are multiple benefits in the use of nanoparticles for ocular drug delivery; for example, the nanometer scale allows for increased permeability across the blood-aqueous barrier. They also allow better drug stability in the formulation and sustained drug delivery [90].

Nanoparticles are able to deliver drugs either by having the active ingredient adsorbed or encapsulated on the surface, or by having it incorporated into the particle itself [53].

**Figure 5:**
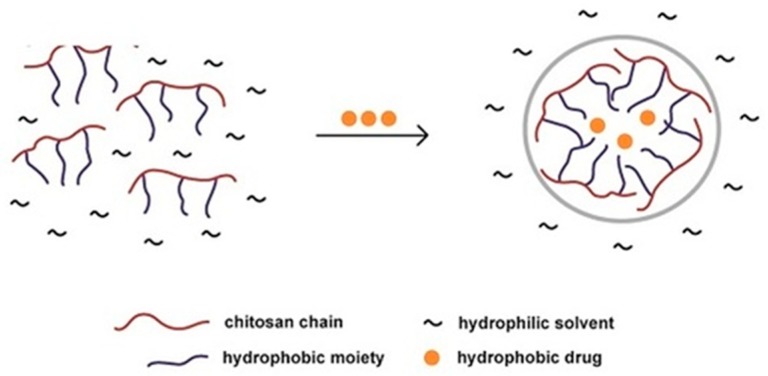
Illustration of hydrophobic drug entrapment within a chitosan nanoparticle, within a hydrophilic solvent environment [46].

The figure below (Figure 5) demonstrates how chitosan is able to form nanoparticles to aid the delivery of hydrophobic drugs in a hydrophilic environment. This is an example of how nanoparticles are able to protect drugs from the solution or environment into which they are placed [46].

In order for nanoparticles to be administered topically to the eye, they need to be incorporated into a suspension system. This system is often designed with a hydrogel as a base [91]. Thus, while nanoparticles aid in the permeation of the drug through the barriers of the eye, the polymeric hydrogel allows for increased mucoadhesion to aid in greater bioavailability. A system such as this has been developed with the incorporation of levofloxacin, which showed increased corneal residence time as well as increased antibacterial activity when compared to that of a levofloxacin solution [92].

A study was performed by Abrego et al., whereby pranoprofen was incorporated into polymeric nanoparticles dispersed within a hydrogel. PLGA was used as the polymer to formulate the nanoparticles and Carbomer 934 (a polyacrylic acid) was selected as the hydrogel. The resulting formulations were compared against a free drug pranoprofen solution and commercial eye drops. The nanoparticle formulations showed prolonged in vitro drug release profiles in comparison to the other two formulations. Although the resulting formulations of this study did not show significant improvement in the corneal permeation of pranoprofen, it is worth noting that the nanoparticle system would be preferred, preventing irritation of the eye, in relation to the the commercial eye drops, which are rapidly cleared from the eye after administration [92].

Although the permeation of molecules into the posterior segment of the eye has been shown to be incredibly low, the development of nanoparticle containing systems, which are administered into the posterior segment of the eye through intravitreal or subretinal injections, will allow for increased mucoadhesion and sustained drug delivery. This in turn decreases the frequency that the injection needs to be administered, resulting in a decrease in side effects often associated, such as retinal detachment, hemorrhage or cataract development [1].

### 5.3. Nanosuspensions

Nanosuspensions are made of a drug dispersed through a colloidal carrier which is within the nanometer range. These systems are usually stabilized by the presence of a polymer or surfactant and allow for increased retention at the cornea as well as improved bioavailability of ocular active ingredients, such dexamethasone. They have also been shown to increase the antibacterial activity [93].

Nanosuspensions have been considered as a possible delivery system for hydrophobic drugs. They allow for easy administration for the patient in the form of a drop, as well as the benefits of increased residence time and bioavailability [45,94].

Polymeric nanosuspensions have been investigated for the delivery of ocular active ingredients because they harness the biocompatibility benefit of polymers, meaning they can be applied to the eye topically without causing irritation to the cornea [95]. A variety of polymers, such as polycaprolactone and poly(lactic-co-glycolic acid), have been investigated for inclusion in a nanosuspension drug delivery system as they are easy to prepare [75]. These drug delivery systems also offer a prolonged drug release profile, compared to that of an aqueous solution [96].

This property of nanosuspensions was demonstrated in a study conducted by Pignatello et al. The researchers formulated a nanosuspension designed for the intraocular delivery of ibuprofen. The formulation was comprised of a Eudragit RS100^®^ nanoparticulate suspension. In vivo studies showed that the concentration of ibuprofen measured in the aqueous humor was significantly higher in the rabbits treated with the nanoparticulate suspensions, than in those treated with a ibuprofen solution of the same concentration (1.54 ± 0.06 µ·mL^−1^ compared to 0.93 ± 0.08 µ·mL^−1^ respectively). This was attributed to the fact that the sustained drug release profile, which was observed in the nanoparticulate suspension, allowed for better penetration through the ocular barriers, ensuring that a higher concentration of drug reaches the anterior chamber [97].

### 5.4. Nanomicelles

Nanomicelles are a form of nanotechnology composed of amphiphilic monomers. These polymers are able to self-assemble into micelles ranging from 20–200 nm. The ability of a polymer to self-assemble into micelles is dependent on its concentration within the solution. The critical micelles concentration (CMC) must be reached in order for micelles to form [98]. The amphiphilic nature of the polymers arranges with a hydrophobic core and a hydrophilic outer layer. This allows for the encapsulation and transport of poorly water-soluble molecules [98,99].

Polymeric micelles, when used in ocular drug delivery, are able to encapsulate the active ingredient and deliver it to the appropriate target site. This helps to alleviate some of the localized adverse side effects that are experienced when using eye drops, such as dry eyes, burning sensations or stinging [98]. It also protects the active ingredient from degradation and increases its permeation through the epithelial layers [100]. Polymeric nanomicelles also offer benefits in terms of preparation and formulation, having a low CMC and being stable in solution [98].

Nanomicelles, while providing a number of benefits for formulations which treat the anterior segment of the eye, have also been investigated to aid in drug delivery to the posterior segment, without the need for invasive injections. This has been shown to be possible in the delivery of rapamycin to the posterior segment of the eye after topical administration [101].

A nanomicellar formulation was developed by Cholkar et al., and studied from the delivery of rapamycin to the posterior segment of the eye. Rapamycin is hydrophobic, poorly soluble and is both a pH and light sensitive drug. The development of a nanomicellar fornulation was designed to assist in overcoming these challenges through a non-invasive delivery route. A blend of materials was employed; namely tocopherol, polyethylene glycol succinate-1000, octoxynol-40 (with a viscosity enhancer), as well as povidone K 90; employed to overcome the rapid drainage of the formulation following topical application. An in vivo distribution study was performed which illustrated that the formulation was able to deliver rapamycin to the posterior segment of the eye after it had been applied topically. The results of the study showed that rapamycin was retained within the retina-choroid at a concentration of 362.35 ± 56.7 ng/g of tissue. This research provides a possible method of back-of-the-eye drug delivery, without using invasive procedures such as intravitreal injections [100].

### 5.5. Nanofibers

Nanofibers are made through one of two processes, electrospinning or sol-gel process. They offer an advantage of a large surface area (nanofibers with a diameter of 100 nm can offer a surface area of up to 1000 square meters per gram). This allows for increased drug loading abilities [102,103]. This, coupled with the sustained release profiles of biodegradable polymers as discussed earlier, may provide a new innovation in the treatment of posterior section conditions, such as age-related macular degeneration. The process of electrospinning allows for the easy incorporation of active ingredients into the system, allowing for increased drug loading within the nanofibers [104]. This process of producing nanofibers also forms a high porous network of fibers [75,105].

In a recent study by Lancina et al., nanofibers were loaded with brimonidine tartrate and investigated as a possible new topically administered formulation for the treatment of glaucoma. Polyamidoamine dendrimers were used for the formulation of the nanofibers. When evaluating the in vivo efficacy of this formulation, researchers found that the dendrimer nanofiber formulation did not cause a drop in intraocular pressure that was significantly expected [106].

### 5.6. Nanoliposomes

Nanoliposomes (including solid lipid nanoparticles and nanostructured lipid carriers) offer some interesting benefits in terms of ocular drug delivery. In addition to improving corneal permeability and residence time, they are able to withstand autoclave sterilization and can be loaded with poorly water-soluble drugs. This allows for the development of formulations that contain active ingredients which have a low bioavailability when administered as a suspension, such as penicillin G [2]. Liposomes, with a lipid outer layer and an aqueous core, are able to be loaded with either lipophilic active (in the outer layer), hydrophilic (in the core) or amphiphilic active ingredients [107,108].

Nanoliposomes have also been shown to slow the rate of clearance of the formulation. They have been investigated for drug delivery to both the anterior and posterior segments of the eye. An important consideration for a drug delivery system that contains nanoliposomes is that it is prepared as a homogenous formulation to prevent the formation of agglomerates which could cloud the vitreous [107]. A recent study developed nanoliposomes in a formulation for the treatment of dry eye disease (DED). The incorporation of nanoliposomes allowed for the formulation to more closely resemble the make-up of the natural tears [109].

In a recent study by Wang et al., nanoliposomes were investigated for the ocular delivery of brinzolamide (an anti-glaucoma active ingredient), as a complex with hydropropyl-β-cyclodextrin. This drug delivery system was formulated and compared to a commercially available brinzolamide suspension. While the nanoliposome formulation displayed only a moderate sustained release profile, it showed a 9.36-fold increase in the permeability coefficient. The nanoliposome formulation (with a brinzolamide concentration of 1 mg/mL) also showed an improved intraocular pressure (IOP) reduction in comparison to the commercial product (with a brinzolamide concentration of 10 mh/mL). Over an extended period of time; the nanoliposome formulation maintained an effective IOP until the 12th hour, whereas the suspension reached its peak IOP reduction at 1 h [108].

### 5.7. Nanowires

Nanowires are structures that have a diameter within the nanoscale range but are also able to have an elongated length. This allows for increased drug loading over other nanostructures such as nanoparticles [110]. They have often been combined with drug particles in drug delivery systems which were then able to adhere to epithelial tissues [111]. Nanowires are able to offer controlled drug release profiles [112]. This property would be beneficial in the delivery of drugs to the eye as it will reduce the frequency at which formulations, such as intravitreal injections, need to be administered.

In a recent study by Christiansen et al., poly(ɛ-caprolactone) (PCL) short nanowires, PCL electrospun and PCL smooth scaffolds, were compared for the delivery of retinal progenitor cells (RPCs). RPCs are used as a restorative procedure for retinal degenerative conditions, due to retinal cells not being able to regenerate. The PCL short nanowires provided a delivery system that displayed appropriate stiffness and flexibility, was able to be inserted into the retina effectively, as well as being flexible enough not to distort the shape of the subretinal space. The nanowires also provided the highest preservation rate of the overlying retina when compared to the other two scaffolds (17% higher than the PCL electrospun and 25% greater than the PCL smooth scaffold system) [113].

## 6. Future Developments in Ocular Drug Delivery Using Biodegradable Polymers and Nanotechnology

As of 2018, there were 51 nanotechnology medical products on the market, as reported by Patra et al. The researchers also reported a number of nanotechnology-based systems that are in the stage of clinical trials. This shows that the development of nanotechnology is on the rise [114].

**Figure 6:**
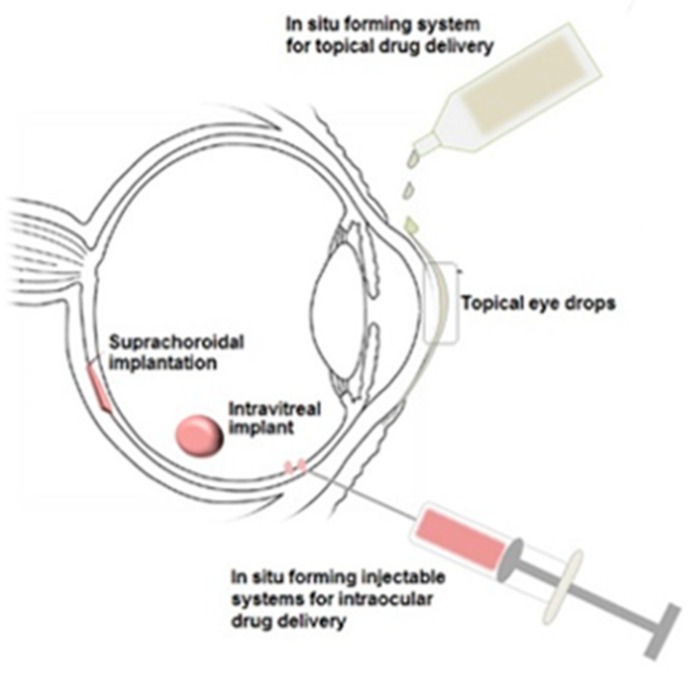
Illustration of in situ gelling systems that could be delivered to the eye employing stimuli-responsive polymers as biodegradable drug delivery platforms [116].

Although polymer-based drug delivery has been the focus of much research in recent years, there are still advances to be made in order to fully enhance the beneficial properties that these materials possess. For example, further investigation into stimuli-responsive polymers could provide many new opportunities for improved ocular drug delivery systems [115]. Figure 6 shows possible administration routes for in situ forming gelling systems to the eye.

Both polymeric drug delivery systems and nanotechnology can be employed in future research for the delivery of larger molecules, such as proteins and peptides, to the eye [116].

Many of the studies that were mentioned in this review stated that, although the formulations developed were successful in terms of in vitro and in vivo studies, they require further investigations and clinical trials in order to be commercially viable. It can also be noted that some formulations, such as nanoparticles and hydrogels, are more extensively researched than others, such as nanowires. These can be attributed to research facility limitations, as well as high costs of undertaking such studies. Thus, further research should be done into these less investigated drug delivery systems, providing greater expansion to the field of biodegradable polymeric nanosystems for ocular drug delivery.

## 7. Conclusions

The importance of a patient’s sight cannot be overstated, and there is a myriad of conditions that places this valuable sense in jeopardy. Although there are many ophthalmic formulations available on today’s market, the drawbacks that they entail, such as low bioavailability and rapid clearance from the eye, leads to below optimum treatment plans for the patient. Patients either have to administer treatment at a regular basis, which puts them at risk for side effects, or undergo frequent, invasive procedures. Biodegradable polymers, together with nanosystems, offer many exciting opportunities in terms of ocular drug delivery. Not only do they provide mechanisms in order to optimize the products currently on the market, they also allow for the development of many new formulations. The polymers that have been highlighted in this article have been shown to be biocompatible, biodegradable and mucoadhesive; all properties that are vital in overcoming the challenges faced by ocular drug delivery. Nanotechnology is rapidly emerging in the field of drug delivery. It has been widely used for diagnosis and treatment in other areas of the body. The advancements that are being made in this field provide a range of drug delivery designs, each offering a unique set of benefits. Innovations are being made in the field of ophthalmic drug delivery, as well as how both polymers and nanosystems can be used in further formulation developments. Thus innovations made in terms of polymeric nanosystem drug delivery are vital for overcoming the challenges faced in treating ocular conditions.

## Figures and Tables

**Table 1:** Advantages and disadvantages experienced by current conventional ocular drug delivery systems.

Dosage Form	Advantages	Disadvantages	References
Eye Drops (Solutions and Suspensions)	Ease of administration. Little discomfort to the patient. Non-invasive.	Low bioavailability. Limit to dosage size. Frequent administration. Low patient adherence.	[2,20,21,25,26]
Ointments	Ease of administration. Decreased clearance rate following administration. High bioavailability than liquid formulations.	Blurred vision. Inaccurate dosing. Challenges in formulating.	[20,28]
Intravitreal Injections	Drug administered directly to posterior segment.	Invasive. Multiple risks with frequent administrations. Frequent administration.	[29,30]
Intraocular Implants	Extended drug release. If biodegradable polymers used, no need for removal. Able to be developed as stimuli-responsive.	Surgical implantation and removal if not biocompatible. Risks associated with insertion. Insertion uncomfortable for patients.	[29,31]
Contact Lenses	Increased residence time compared to other formulations. Keeps drug in contact with the surface of the eye for improved permeation.	Rapid release of drug within the first few hours. Cannot be used continuously. Risk of infection.	[32,33]
Emulsions	Improved bioavailability over other formulations. Improved residence time. Sustained drug release profiles.	Susceptible to flocculation and instability. Destabilized by tear fluid.	[35,36]
